# Patients’ experience of service quality in government and private hospitals in the Qassim Region, Kingdom of Saudi Arabia

**DOI:** 10.25122/jml-2023-0184

**Published:** 2023-11

**Authors:** Mohammad Faleh Alharbi

**Affiliations:** 1 Department of Health Administration, College of Public Health and Health Informatics, Qassim University, Saudi Arabia

**Keywords:** quality, patients experience, health, Saudi Arabia

## Abstract

Patients’ experience of the quality of healthcare services has gained momentum during the last few decades. This study aimed to assess and compare the experience of patients on the service quality in government and private hospitals in the Qassim region of the Kingdom of Saudi Arabia. A cross-sectional online survey was conducted using a Service Quality (SERVQUAL) questionnaire translated into the Arabic language to collect the responses from 381 patients who were hospitalized at government and private hospitals in the Qassim region from April 1, 2022, to March 31, 2023. The patients’ overall satisfaction with the service quality showed that there were significant differences among government and private hospitals. Although the study revealed positive experiences for patients on all dimensions of service quality in both types of hospitals, private hospitals scored a higher value than government hospitals in all the dimensions of service quality. Patients’ feedback on service quality can assist hospital management in identifying key quality issues encountered by the patients during their treatment and rectifying those problems to improve overall healthcare quality and thus patients’ satisfaction.

## INTRODUCTION

The healthcare system in the Kingdom of Saudi Arabia (KSA) has undergone significant progress over time, leading to greater access to healthcare services across the nation. Over the past few decades, a substantial investment was made in the health sector, which has largely contributed in building up a huge healthcare infrastructure including development of health workforce [1]. The health system in KSA currently delivers services through three major sources: (1) health facilities owned by the Ministry of Health (MOH) offer services free at the point of delivery to all Saudi citizens and expatriates employed in the government sector, including their dependents; (2) other government ministries and agencies, such as the armed forces, national guard, ministries of interior and human resources, the Royal Commission of Jubail and Yanbu, and public undertakings, like Arabian American oil company (ARAMCO), provide health coverage to their employees and dependents; and (3) employers in the private sector provide health insurance to all of their workers, including Saudis and expatriates and their dependents to cover the healthcare costs in private facilities. The MOH is the key provider of health services with 287 hospitals (with 45,330 beds) and 2,212 primary healthcare centers across the Kingdom. Other government ministries and public entities operate 51 hospitals to deliver health services to their employees and dependents. The private sector currently operates 159 hospitals (with 17,889 beds) and 3,732 medical complexes to deliver health services across the Kingdom [2].

Over the past few decades, patients’ knowledge and awareness of the quality of services delivered in different health systems have improved significantly [3, 4]. As a result, healthcare providers are now focused on how to improve the quality of care at healthcare institutions. Rapid technological advancements in health care have had an impact on the quality of healthcare services and patients’ experience of the quality of their care [5]. Patients’ experience of service quality is a critical factor for improving the quality of hospital services, as it reveals the extent to which the providers meet patient expectations [6-11]. Patients’ experience with regards to whether the service quality offers good value for their money is influenced by enhancing access, comprehensiveness of services, safety, and equity [12]. In the KSA, the history of quality improvement dates back to the 1990s when the National Committee on Quality Assurance in primary health care was introduced [13]. At the national level, improvements in quality awareness in the government and private sectors, including health care, culminated with the establishment of the King Abdulaziz Quality Award in 1999. This award was aimed at motivating the government and private institutions to provide higher-quality services, including health care.

The Qassim region is one of the 13 provinces in the KSA, located in the geographic core of the Arabian Peninsula. It is home to about 1.4 million people and spans an area of 72,002 square km accounting for 3.2% of the KSA. Almost 57% of population is male and 43% is female with a median age of 26.3 years [2, 14].

Saudi Arabia’s Vision 2030 aims to deliver high-quality health services to the population through privatization. The goal of Vision 2030 and the Health Transformation Program is to improve access to quality health services without incurring any financial burden by providing health insurance to the entire population. Although government and private hospitals deliver various services, there are disparities in their quality, as well as in patients’ satisfaction, waiting times for consultations and surgeries, ease of making appointments, and access to care [15-16]. Studies comparing service quality at government and private hospitals in the KSA are limited, and none exist in the scholarly literature that assess the differences in service quality in government and private hospitals as perceived by the patients in the Qassim region. Therefore, the purposes of this study were to assess and compare how satisfied patients are with the quality of services offered in both government and private hospitals in the Qassim region of the KSA.

## MATERIAL AND METHODS

The current research used a cross-sectional design to assess patients’ experience of the quality of health care delivered in government and private hospitals in the Qassim region of the KSA. Data for the study were collected through an online survey. Patients’ experience of service quality provided in public and private hospitals was measured using Service Quality (SERVQUAL), as an internationally accepted dimensional model.

The framework for measuring the service quality from the users’ perspectives was identified in 1985. This was the SERVQUAL dimensional model, consisting of five dimensions that incorporate clients’ satisfaction: reliability, assurance, tangibility, empathy, and responsiveness [16]. However, this model sparked a debate among many authors who argued that these dimensions can be merged into core and augmented services [17]. In contrast, others voiced doubts due to an overdependence on consumers and a lack of objective measurements [18]. Despite these arguments, researchers worldwide used the SERVQUAL model to assess the quality of the services delivered by various industries [19].

Having already been proven in its effectiveness and quality, the SERVQUAL questionnaire was considered a suitable tool for measuring service quality [20-21].

In this study, all of the government and private hospitals (21 government hospitals and four private hospitals) in the Qassim region of the KSA were included. The participants were all patients hospitalized in a government or private hospitals between the 1^st^ of April 2022 and the 31^st^ of March 2023. Patients who were hospitalized outside the Qassim region were not included in the study. The questionnaire was translated into Arabic and distributed online to all eligible patients in April and May 2023. The questionnaire consisted of 28 items, categorized into seven socio-economic items, four items each on five SERVQUAL dimensions, and one last item on patients’ overall satisfaction with the quality of the services they received. The participants’ responses were collected using a 5-point Likert scale with 1 being “I don’t agree at all” and 5 being “I completely agree” (appendix-1).

Appendix

Of the 450 completed questionnaires, only 381 were considered, as the remaining responses were not related to hospitalization during the selected period of the study. All completed questionnaires were verified for completeness and validated, coded, and entered into a computer. The Statistical Package for the Social Sciences (SPSS) software version 26 was used for data analysis. Cronbach’s alpha was used to test the reliability of the study tools, and the chi-squared test was used to assess the significance of the associations between socioeconomic characteristics and outcome variables (government *versus* private hospitals). The mean difference between government and private hospitals on five quality dimensions and the overall satisfaction of patents were measured through an independent sample t-test.

## RESULTS

Out the 381 participants, 217 (56.95%) were hospitalized in government hospitals and 164 (43.05%) in private hospitals. Descriptive statistics are shown in Table 1, including nationality, gender, age, area of residence, marital status, level of education, and occupation. Among these participants, 92.7% were Saudi nationals, 66.9% were male, 73.2% were below the age of 30 years, 24.7% were between 30 and 50 years of age, and a majority (79%) were living in the urban areas.

**Table 1:** Socioeconomic characteristics of respondents

Variables		Government Hospitals (N=217) N (%)	Private Hospitals (N=164) N (%)	Total (N=381) N (%)	Bivariate statistics (p-value)
Nationality	Saudi	198 (91.2)	155 (94.5)	353 (92.7)	0.226
	Non-Saudi	19 (8.8)	9 (5.5)	28 (7.3)	
Gender	Male	134 (61.7)	121 (73.8)	255 (66.9)	0.013
	Female	83 (38.3)	43 (26.2)	126 (33.1)	
Age	<20 years	125 (57.6)	85 (51.8)	210 (55.1)	0.0161
	20-30	38 (17.5)	31(18.9)	68 (18.1)	
	31-40	29 (13.4)	19 (11.6)	48 (12.6)	
	41-50	19 (8.6)	27 (16.5)	46 (12.1)	
	Above 50	6 (2.8)	2 (1.2)	8 (2.1)	
Area	Urban	164 (75.6)	137 (83.5)	301 (79.0)	0.059
	Rural	53 (24.4)	27 (16.5)	80 (21.0)	
Marital status	Single	102 (47.0)	84 (51.2)	186 (48.8)	0.716
	Married	109 (50.2)	76 (46.3)	185 (48.6)	
	Widow/Widower	6 (2.8)	4 (2.4)	10 (2.6)	
Education	Primary	5 (2.3)	5 (3.1)	10 (2.5)	0.964
	Middle	7 (3.2)	4 (2.4)	11 (2.9)	
	Secondary	43 (19.8)	32 (19.5)	75 (19.7)	
	College	138 (63.6)	107 (65.2)	245 (64.3)	
	Post-graduate	24 (11.1)	16 (9.8)	40 (10.5)	
Occupation	Student	66 (30.4)	42 (25.6)	108 (28.3)	0.052
	Government sector	62 (28.6)	59 (35.9)	121 (31.8)	
	Private sector	20 (9.2)	25 (15.2)	45 (11.8)	
	Retired	69 (31.8)	38 (23.2)	107 (28.1)	

As shown in Table 1, 48.8% of the respondents were single, 48.6% were married, and 2.6% were either a widow or a widower. The majority of them were college graduates (64.3%) and 10.5% were post-graduates. By occupation, 31.8% had government jobs, 11.8% had private jobs, 28.7% were retired from service, and 28.3% were students.

The reliability of the five dimensions of quality—tangibility, reliability responsiveness, assurance, and empathy—was tested using Cronbach’s alpha. All dimensions were reliable and values of α varied between 0.849 for tangibility and 0.912 for assurance, which shows that all items under each dimension were consistent and coherent (Table 2).

**Table 2:** Reliability measures of service quality dimensions

Dimensions	Factor Loading
**Tangibility (α=0.849)**	
Hospital equipment is modern and up to date.	0.645
Hospital physical facilities are visually appealing.	0.909
Health service providers are neat in their appearance.	0.660
Materials related to the services are physically available.	0.607
**Reliability (α=0.893)**	
Hospital provides services without much waiting time.	0.617
Health service providers are keen about resolving health problems.	0.675
The hospital always gives accurate results at the first instance.	0.767
The hospital maintains error-free records/reports.	0.569
**Responsiveness (α=0.858)**	
Hospital personnel inform you exactly when services will be provided.	0.523
Hospital personnel give you services promptly.	0.648
Hospital personnel are always ready to help you.	0.662
Hospital personnel are available to respond to your requests.	0.501
**Assurance (α=0.912)**	
Hospital personnel’s behavior inspires confidence in you.	0.679
You feel a sense of security in your dealings with hospital personnel.	0.739
Hospital personnel are reliably polite with you.	0.685
Hospital personnel have adequate knowledge to respond to your questions.	0.660
**Empathy (α=0.906)**	
Hospital personnel provide you with individual attention.	0.741
Hospital has convenient operating hours for all patients.	0.541
Hospital has your best interests at heart.	0.738
Hospital personnel understand your specific health needs.	0.703

**Figure 1:**
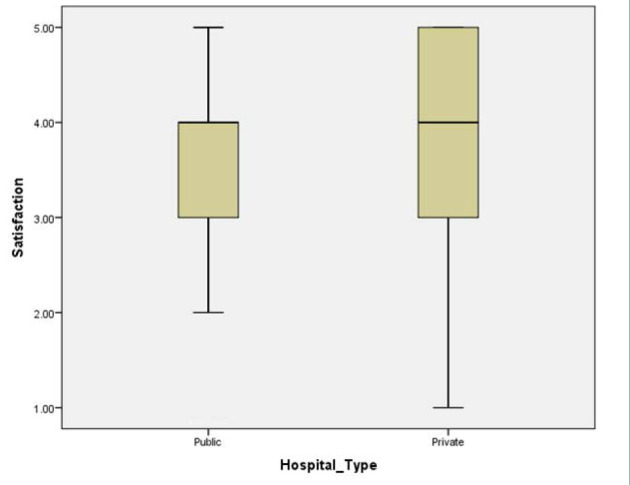
Satisfaction scores of patients between government and private hospitals

This study indicates a difference in patients’ experience of service quality between hospitals in the government and private sectors. Table 3 shows a significant difference between government and private hospitals in all five service quality dimensions (at the 0.01 level), except for responsiveness, where the difference was significant at the 0.05 level. Overall satisfaction between government and private hospitals also showed a significant difference among patients at the 0.05 level (Figure 1).

**Table 3:** Patients’ perceived service quality in government and private hospitals

Dimensions	Mean (SD)		Mean difference	F-Value
	Government hospital	Private hospital		
Tangibles	3.75 (0.819)	4.011 (0.932)	- 0.258	4.326***
Reliability	3.54 (0.980)	4.05 (0.991)	-0.516	5.505***
Responsiveness	3.40 (0.942)	3.94 (1.050)	-0.538	2.036**
Assurance	3.71 (0.904)	4.115 (0.905)	-0.395	3.399***
Empathy	3.37 (0.92)	3.86 (1.06)	-0.490	3.715***
Satisfaction	3.611 (1.08)	3.87 (1.19)	-0.261	4.471**

This study also revealed that for each dimension of service quality, private hospitals scored a higher value than government hospitals. Therefore, patients treated in private hospitals reported higher levels of satisfaction than those in government hospitals.

## DISCUSSION

The objective of this research was to assess patients’ experience of the service quality provided by government and private hospitals in the Qassim region of the KSA. A comparison of means for each dimension makes it abundantly evident that, overall, patients in both public and private hospitals had a positive experience with the services provided. This is similar to the findings of a study conducted in the eastern region of the KSA [22]. This finding is also supported by a recent study on the comparison of patients’ satisfaction toward healthcare performance among private and government hospitals in the KSA [23].

Earlier assessments of patients’ experience of service quality in government and private hospitals have yielded mixed results. A study in the United Arab Emirates (UAE) using the dimensions of empathy, tangibility, reliability, responsiveness, and skills found that government hospitals scored better than the private sector in all dimensions [24]. However, similar studies in Turkey conducted in 2006 and 2010 indicated that the quality of services in private hospitals was higher than in government hospitals [25-27]. Similar findings were also supported by studies conducted in Pakistan [28-29].

The study showed that patients who had inpatient treatment in government hospitals were satisfied in terms of all dimensions of service quality primarily because the public health system in the KSA is fully government-funded and provided free of cost to its citizens. This allows for better access to specialized care, which explains the better score for government hospitals [22]. Since the launch of Vision 2030, the health system in the KSA has shown rapid improvements in terms of access to quality care [30]. As a result of increased competition in the health-care industry, both government and private hospitals are compelled to deliver a higher quality care, making it more difficult to satisfy patients’ expectations [31-32].

The majority of the participants in this study are Saudi men who are married, live in urban areas, and have a bachelor’s degree. The results also showed that young adults are the largest users of healthcare services, and more than 73% of respondents were below 30 years of age. The mean service quality delivered in government and private hospitals varies considerably across all five dimensions, with health services in private sector scoring higher across the board. This finding was corroborated by earlier studies in Pakistan [33], Turkey [25, 27], India [34], and Indonesia [35, 36]. This finding suggests that private hospitals offer healthcare services of higher quality than government hospitals in all dimensions of quality of care. A recent research in the KSA also showed that patients who sought treatment in a private hospital were highly satisfied with the behavior, attitude, and patient-provider communications compared with similar treatments in government hospitals [22]. Private hospital administration is shaped by competing demand to maximize efficiency and to focus on marketing their services to generate more profits. In contrast, in the government sector, political and administrative pressures play more significant roles in improving the quality of care [23].

Reliability tests confirmed the consistency and stability of the service quality dimensions. Additionally, the independent sample t-test shows that the results may be generalized to the population in the region. The study also shows a statistically significant difference in the overall satisfaction of patients in the government and private hospitals in the Qassim region.

However, the experience of patients on dimensions of “empathy” and “responsiveness” in both government and private hospitals were lower compared to other dimensions. The experience of government hospitals was lowest for “empathy” (3.37) and “responsiveness” was the second lowest (3.40). These findings suggest that government hospitals need to focus more on the dimensions of empathy and responsiveness. The experience of “empathy” in government hospitals should be improved by giving more attention to areas such as individual attention to patients, convenient operating hours, work in the best interest of patients and understand the specific needs of patients. The experience of “responsiveness” in government hospitals could be improved by focusing on the service requirements of patients, prompter services, a helpful attitude, and always responding to patients’ requests.

It is important to note how government and private hospitals are distributed between urban and rural locations in the region. According to MOH statistics for 2021, there were 25 hospitals in the region, of which 19 are MOH hospitals, two other government hospitals, and four are private hospitals. While MOH hospitals offer free services to all Saudi nationals, and expatriates employed in the government sector including their dependents; expatriates and Saudi nationals working in the private establishments use health services at private hospitals [2]. The current study showed that 43.9% of Saudi nationals used private hospital services, reflecting the significance of the private health sector in the region. Under Vision 2030, the KSA has implemented several policy measures, including interest-free loans to construct hospitals to encourage private sector involvement in the delivery of health services [37]. According to MOH data, inpatient admissions to private hospitals in the KSA were around 1.10 million in 2021, compared to 1.37 million admissions to MOH hospitals [2].

### Limitations of the study

This research is the first attempt in the Qassim region to assess the patients’ experience of service quality in government and private hospitals. However, it is not free from limitations. Firstly, the study might have come across a response bias, which is common in online surveys. Secondly, it is difficult to know if the respondents provided their own responses or were assisted by another person. Thirdly, the study covered a small sample of non-Saudi patients, and cannot be put in the demographic context. Future studies should also consider a larger sample that includes more non-Saudi patients to gain their perception on service quality in public and private hospitals. Fourthly, the study's findings were restricted to the Qassim region, therefore they do not reflect the population of the KSA. Fifth limitation is the use of single item to measure overall satisfaction which may create multivariate issues; therefore, it is recommended to use at least 3-5 items to measure overall satisfaction on quality and report internal consistency in future studies. Finally, the study did not interpret why the private sector performed better than the government hospitals from a cultural point of view. Therefore, future studies may focus on the influence of national culture and Islamic values on the performance level of public and private hospitals in the Kingdom.

## CONCLUSION

Patients’ feedback is extremely important when designing hospital policies since it helps improve the service quality in healthcare institutions. The results of this research will assist the administrators of government and private hospitals in the KSA in identifying the major quality-related issues encountered by the patients and rectify those problems to improve overall health-care quality and patients’ satisfaction. The study showed that private hospitals in the KSA offer healthcare services of a higher quality than government hospitals in all quality dimensions and are more adaptable to the changing environment and turning them into a competitive advantage. The study’s findings will also help government hospitals in improving their service quality and, consequently, improve patients’ overall satisfaction.

## Data Availability

Further data is available from the corresponding author on reasonable request.
