# Supplementary material for: Patients’ experience of service quality in government and private hospitals in the Qassim Region, Kingdom of Saudi Arabia
Source: J Med Life. 2023 Nov;16(11):1622–7. doi: 10.25122/jml-2023-0184 (PMC10893560; doi:10.25122/jml-2023-0184)
Supplement: Supplementary file 1 [file JMedLife-16-1622-s001.pdf]

Appendix

MEASURING SERVICE QUALITY IN PUBLIC AND PRIVATE HOSPITALS – A STUDY IN QASSIM REGION, SAUDI ARABIA

Respondents' Profile

1	Nationality:	Saudi
		Non-Saudi
2	Gender:	Male
		Female
3	Age:	< 20 years
		20-30 years
		31-40 years
		41-50 years
		51- 60 years
		Above 60 years
4	Area:	Urban
		Rural
5	Marital Status:	Single
		Married
		Divorced
		Widowed
6	Occupation:	Student
		Government sector
		Private sector
		Retired
7	Educational level:	Illiterate
		Elementary/intermediate
		Secondary school
		Diploma
		Bachelor
		Master and above

HOW FAR DO YOU AGREE/DISAGREE WITH THE FOLLOWING STATEMENTS ON 5-POINT SCALE?

Strongly Disagree	Disagree	Neutral	Agree	Strongly Agree
1	2	3	4	5

Tangibility

1	The hospital has modern-looking equipment	1	2	3	4	5
2	The physical facilities in the hospital are visually appealing	1	2	3	4	5
3	Personnel in the hospital are neat in appearance	1	2	3	4	5
4	Materials associated with the service (such as pamphlets or statements) are visually appealing	1	2	3	4	5

Licensed under CC BY 4.0

Appendix

Reliability

1	When the hospital promises to do something by a certain time it does so	1	2	3	4	5
2	When you have a problem, the hospital shows a sincere interest in solving it	1	2	3	4	5
3	The hospital gets things right the first time	1	2	3	4	5
4	The hospital insists on error-free records	1	2	3	4	5

Responsiveness

1	The personnel in the hospital tell you exactly when services will be performed	1	2	3	4	5
2	Personnel in the hospital gives you prompt service	1	2	3	4	5
3	Personnel in the hospital are always willing to help you	1	2	3	4	5
4	Personnel in the hospital are never be too busy to respond to your requests	1	2	3	4	5

Assurance

1	The hospital gives you individual attention	1	2	3	4	5
2	The hospital has operating hours convenient to all its patients	1	2	3	4	5
3	The hospital has your best interests at heart	1	2	3	4	5
4	The personnel of the hospital understand your specific needs	1	2	3	4	5

Overall Satisfaction

1	Overall satisfaction with the health services received	1	2	3	4	5

Licensed under CC BY 4.0
